# Subclinical cardiotoxicity following adjuvant dose-escalated FEC, high-dose chemotherapy, or CMF in breast cancer

**DOI:** 10.1054/bjoc.1999.0998

**Published:** 2000-02

**Authors:** T Erselcan, K J A Kairemo, T A Wiklund, M Hernberg, C P Blomqvist, M Tenhunen, J Bergh, H Joensuu

**Affiliations:** Department of Oncology, Helsinki University Central Hospital, Haartmaninkatu 4, PO Box 180, Hyks, Helsinki, FIN-00029, Finland; Department of Oncology, Karolinska Hospital, PO Box 60500, Stockholm, SE-104 01, Sweden

**Keywords:** breast cancer, chemotherapy, adverse effects, high-dose therapy, epirubicin, antimyosin scintigraphy

## Abstract

We compared adjuvant chemotherapy-related myocardial damage by antimyosin scintigraphy in patients who received either nine cycles of FEC (fluorouracil, epirubicin and cyclophosphamide) where the doses of epirubicin and cyclophosphamide were escalated according to the leucocyte nadir (group I, *n* = 14), three cycles of FEC followed by high-dose chemotherapy with alkylating agents (CTCb) given with the support of peripheral blood stem cell transplantation (group II, *n* = 14), or six cycles of standard intravenous CMF (cyclophosphamide, methotrexate and fluorouracil; group III, *n* = 8). The cardiac uptake of In-111-antimyosin-Fab (R11D10) antibody was measured and the heart-to-lung ratio (HLR) calculated 8–36 months after the last dose of chemotherapy. Cardiac antimyosin antibody uptake was considerably higher among patients treated with nine cycles of dose-escalated FEC than among those who were treated with three cycles of FEC and high-dose CTCb (HLR, median 1.98; range 1.36–2.24 vs median 1.51; range 1.20–1.82;*P*< 0.001), or those treated with CMF (median 1.44; range 1.15–1.68;*P*< 0.001). The difference between groups II and III was not significant (*P*> 0.1). A linear association was found between the cumulative dose of epirubicin and the cardiac antimyosin uptake (*P*< 0.001). We conclude that subclinical cardiac damage caused by three cycles of conventional-dose FEC followed by one cycle of high-dose CTCb chemotherapy is small as compared with the damage caused by dose-escalated FEC. © 2000 Cancer Research Campaign


*British Journal of Cancer* (2000) **82**(4), 777–781
© 2000 Cancer Research Campaign
DOI: 10.1054/ bjoc.1999.0998, available online at http://www.idealibrary.com on IDEAL®

### Subclinical cardiotoxicity following adjuvant dose-escalated FEC, high-dose chemotherapy, or CMF in breast cancer

**T Erselcan[1], KJA Kairemo[1], TA Wiklund[1], M Hernberg[1], CP Blomqvist[1], M Tenhunen[1], J Bergh[2] and H Joensuu[1]**

[1]Department of Oncology, Helsinki University Central Hospital, Haartmaninkatu 4, PO Box 180, Hyks, FIN-00029 Helsinki, Finland; [2]Department of Oncology, Karolinska Hospital, PO Box 60500, SE-104 01 Stockholm, Sweden

**Summary** We compared adjuvant chemotherapy-related myocardial damage by antimyosin scintigraphy in patients who received either nine cycles of FEC (fluorouracil, epirubicin and cyclophosphamide) where the doses of epirubicin and cyclophosphamide were escalated according to the leucocyte nadir (group I, $n = 14$), three cycles of FEC followed by high-dose chemotherapy with alkylating agents (CTCb) given with the support of peripheral blood stem cell transplantation (group II, $n = 14$), or six cycles of standard intravenous CMF (cyclophosphamide, methotrexate and fluorouracil; group III, $n = 8$). The cardiac uptake of In-111-antimyosin-Fab (R11D10) antibody was measured and the heart-to-lung ratio (HLR) calculated 8–36 months after the last dose of chemotherapy. Cardiac antimyosin antibody uptake was considerably higher among patients treated with nine cycles of dose-escalated FEC than among those who were treated with three cycles of FEC and high-dose CTCb (HLR, median 1.98; range 1.36–2.24 vs median 1.51; range 1.20–1.82; $P < 0.001$), or those treated with CMF (median 1.44; range 1.15–1.68; $P < 0.001$). The difference between groups II and III was not significant ($P > 0.1$). A linear association was found between the cumulative dose of epirubicin and the cardiac antimyosin uptake ($P < 0.001$). We conclude that subclinical cardiac damage caused by three cycles of conventional-dose FEC followed by one cycle of high-dose CTCb chemotherapy is small as compared with the damage caused by dose-escalated FEC. © 2000 Cancer Research Campaign

**Keywords:** breast cancer; chemotherapy; adverse effects; high-dose therapy; epirubicin; antimyosin scintigraphy

Anthracyclines are used extensively in therapy of several types of human cancer where long-term survival and cure are common, such as haematological malignancies and paediatric cancers. They are now increasingly used also in adjuvant therapy of breast cancer, because the results of some trials suggest that anthracycline-containing combinations are associated with better survival than non-anthracycline-containing regimens (Early Breast Cancer Trialists' Collaborative Group, 1998). These trials have, however, only limited follow-up of a few years. Cardiotoxicity is a well-established side-effect of anthracyclines, and subclinical cardiotoxicity of adjuvant and other curative therapies might manifest only several decades after treatment when the myocardial reserves start to decrease in old age. This might counterbalance the short-term absolute survival benefit associated with anthracycline-containing regimens over CMF (cyclophosphamide, 5-fluorouracil and methotrexate), which is only about 3% at 5 years of follow-up according to the meta-analysis (Early Breast Cancer Trialists' Collaborative Group, 1998). Cardiotoxicity of anthracyclines may be reduced by low-peak therapies, such as anthracyclines given as long infusions (Legha et al, 1982). A novel iron-chelating agent, dexrazoxane, has reduced anthracycline-associated cardiotoxity in several randomized trials, and apart from one study, it has not reduced response rates (Green, 1998).

*Received 22 March 1999*
*Revised 22 September 1999*
*Accepted 20 October 1999*

*Correspondence to:* H Joensuu

High-dose chemotherapy is used increasingly in therapy of breast cancer both in the US and Europe (Antman et al, 1997; Grathwohl et al, 1997). In the USA, breast cancer is the most common indication for high-dose chemotherapy given with the support of either bone marrow or peripheral blood stem cell rescue at present (Antman et al, 1997). It is currently highly controversial whether high-dose chemotherapy is superior to conventional-dose chemotherapy either in the adjuvant or the metastatic setting, but several controlled trials are currently in progress to resolve the issue. Favourable results have been obtained with adjuvant high-dose therapy in phase II studies (Peters, 1996), and many breast cancer patients with a high risk of relapse are now given high-dose therapy. However, besides its efficacy, the long-term risks of high-dose therapy are unsettled. Little is known about the subclinical cardiac toxicity related to high-dose adjuvant therapy, which might be of great importance for the long-term survivors from cancer.

Endomyocardial biopsy is the most reliable method to diagnose anthracycline-induced cardiotoxicity. However, a non-invasive method of indium-111 monoclonal antimyosin antibody scintigraphy has been shown to be highly sensitive for detecting cardiac damage. The method is capable for detecting even small areas of myocardial damage or necrosis in a variety of diseases (Estorch et al, 1993; Verna et al, 1995; Astorri et al, 1996; Ballester et al, 1997*a*, 1997*b*; Bengel et al, 1997; Schutz et al, 1997). Moreover, it may be the only non-invasive method currently available for detection of subclinical myocardial damage.

The aim of the present study was to compare subclinical myocardial damage caused by three different adjuvant therapies used in treatment of breast cancer. Two of the regimens contained epirubicin, which is less cardiotoxic than doxorubicin when compared on a milligram per milligram basis, but is usually used in 20–30% larger doses than doxorubin. Patients in one of the groups evaluated received high-dose therapy with CTCb, which is one of the most commonly used high-dose regimens, and we studied also patients who had been treated with a presumably low-cardiotoxic regimen (cyclophosphamide, methotrexate and fluorouracil CMF) as controls.

### PATIENTS AND METHODS

### Patients and therapy given

Thirty-six women with histologically proven breast cancer were entered in the study. None of the participants had any known previous cardiac disease in history, medication for cardiac disease, or signs of cardiac dysfunction in the clinical examination. The renal and hepatic functions were required to be normal. The staging examinations included mammography, chest X-ray, isotope bone scan, ultrasound or computerized tomography of the abdomen and a blood chemistry profile. Patients treated with anthracyclines had an electrocardiogram recorded before starting chemotherapy. Both patients who received the high-dose therapy and those treated with dose-escalated FEC (5-fluorouracil, epirubicin and cyclophosphamide) participated in a randomized trial carried out by the Scandinavian Breast Cancer Group comparing dose-escalated FEC to high-dose chemotherapy in high-risk breast cancer with at least five axillary nodes positive for cancer. The patients had been subjected to modified radical mastectomy or breast conserving surgery prior to adjuvant chemotherapy, and following chemotherapy they received post-operative radiotherapy to the breast, chest wall and the axilla. The total dose was 50 Gy. In patients treated with mastectomy, radiotherapy was given to the chest wall with 6–9 MeV electrons and in patients treated with breast conservation the breast was irradiated with 2 opposed oblique fields to avoid cardiac irradiation. We required that 6–36 months had elapsed from the last dose of adjuvant chemotherapy (range 8–36 months). The patients fulfilling these inclusion criteria were chosen at random for the study from the patients visiting the Department of Oncology, Helsinki University Hospital, for follow-up. The study was approved by an Ethical Committee. A written informed consent was required.

### Chemotherapy regimens

Three types of adjuvant chemotherapy were compared. In group I (dose-escalated FEC, $n$ = 14) the patients received FEC every 3 weeks for nine cycles, supported by granulocyte growth factors (G-CSF, Filgrastim®). The doses of cyclophosphamide and epirubicin were modified according to the nadir leucocyte and platelet counts (Bergh et al, 1998). The epirubicin doses ranged from 60 to 120 mg m$^{-2}$ per cycle (the starting dose was 75 mg m$^{-2}$), the cyclophosphamide doses from 600 to 1800 mg m$^{-2}$ per cycle (the starting dose was 900 mg m$^{-2}$), and the 5-fluorouracil dose was fixed to 600 mg m$^{-2}$. In group II (high-dose chemotherapy, $n$ = 14) first two cycles of the standard FEC (5-fluorouracil 600 mg m$^{-2}$, epirubicin 60 mg m$^{-2}$, cyclophosphamide 600 mg m$^{-2}$) were given 3-weekly, followed by a third FEC cycle with escalated cyclophosphamide (1200 mg m$^{-2}$) with G-CSF for stem cell mobilization, and finally a cycle of high-dose chemotherapy was given. The high dose regimen was CTCb, consisting of cyclophosphamide 6000 mg m$^{-2}$, thiotepa 600 mg m$^{-2}$ and carboplatin 800 mg m$^{-2}$ as a continuous infusion over 4 days, followed by infusion of peripheral blood stem cells. No further cycles of FEC were given after the high-dose therapy. In group III (CMF, $n$ = 8) therapy consisted of standard intravenous (i.v.) CMF (cyclophosphamide 600 mg m$^{-2}$, methotrexate 40 mg m$^{-2}$, 5-fluorouracil 600 mg m$^{-2}$) for six cycles repeated every 3 weeks.

**Table 1** Characteristics of the patients and treatments given

| Characteristic | Dose-escalated FEC × 9 | FEC × 3 +CTCb | CMF × 6 |
|---|---|---|---|
| Median age (range), years | 50 (39–59) | 51 (36–59) | 48 (38–54) |
| Months since last dose of chemotherapy median (range), months | 17 (8–30) | 24 (8–36) | 17 (16–20) |
| Median cardiac dose median (range), Gy | 1.0 (1.0–2.5) | 1.0 (1.0–2.5) | 1.0 (1.0–2.5) |
| Cumulative dose of epirubicin, median (range), mg m$^{-2}$ | 734 (502–895) | 181 (174–183) | None |
| Cumulative dose of cyclophosphamide median (range mg m$^{-2}$) | 9189 (5713–10 800) | 8395 (7829–8670) | 3600 (3000–3600) |

The characteristics of the patients are shown in Table 1. The groups are well balanced with respect of age and time from the last dose of chemotherapy. The cardiac absorbed dose from external radiotherapy was minimal in all patients (median, 1 Gy in all three groups). Only 11 patients received more than a cumulative dose of 1 Gy to the myocardium (group I, $n$ = 3; group II, $n$ = 5; group III, $n$ = 3), and the maximum cumulative myocardial dose in any patient in the series was only 2.5 Gy. Two group I patients, four group II patients and three group III patients were treated with 2 opposed tangential photon fields after breast conserving surgery, whereas the rest received electrons to the thoracic wall following mastectomy. The median cumulative dose of epirubicin was 734 mg m$^{-2}$ among patients treated with escalated FEC and 181 mg m$^{-2}$ in the high-dose group. The median cumulative dose of cyclophosphamide was slightly higher in group I than in group II (9189 and 8395 mg m$^{-2}$ respectively), and clearly less among patients treated with CMF (3600 mg m$^{-2}$).

### Scintigraphy

Scintigraphy was performed 48 h after an i.v. injection of 0.5 mg of monoclonal R11D10-Fab-fragments (Centocor, Leiden, The Netherlands), labelled with 74 MBq of In-111. The diethylenetriaminepentaacetic acid (DTPA)-derivative of this antibody fragment was labelled as described in detail elsewhere (Kairemo et al, 1990). Anterior planar chest scans were obtained using a gamma camera (General Electric, Maxi 500) with a medium energy collimator. A 20% window was centred on both peaks (173 keV and 247 keV) of In-111, and a minimum of 500 000 counts were collected and stored in a 128 × 128 matrix frame. Evaluation of the scans was made blinded for the treatments given and other clinical data. The absolute cardiac antimyosin uptake (% ID cm$^{-3}$) was calculated using a region of interest (ROI) technique with the aid of a cardiac phantom. The heart-to-lung ratio (HLR) was deter-

 © 2000 Cancer Research Campaign

**Table 2** Uptake of In-111-labelled antimyosin antibody in patients treated with three different adjuvant chemotherapy protocols

| Characteristic | Dose-escalated FEC × 9 (group I, $n$ = 14) | FEC × 3 + CTCb (group II, $n$ = 14) | CMF × 6 (group III, $n$ = 8) |
|---|---|---|---|
| Cardiac antimyosin uptake (% ID $cm^{-3}$) | | | |
| Median | 0.014 | 0.009 | 0.009 |
| (range) | (0.008–0.030) | (0.002–0.016) | (0.005–0.016) |
| Heart-to-lung ratio | | | |
| Median | 1.98 | 1.51 | 1.44 |
| (range) | (1.36–2.24) | (1.20–1.82) | (1.15–1.68) |

mined by dividing the average counts per pixel in the heart ROI by the average counts per pixel in the lung ROI. The HLR was used as a semiquantitative parameter for myocardial damage. A value larger than 1.58 was considered as pathological based on the data published earlier by Carrio et al (1993).

### Statistical analysis

Comparisons of different groups was done using the non-parametric Mann–Whitney *U*-test. Spearman's correlation coefficient was used in assessing correlations between two parameters. All *P*-values are two-tailed.

### RESULTS

The cardiac antimyosin uptake and HLRs are shown in Table 2. As expected, the uptake was significantly greater among patients treated with dose-escalated FEC than the women treated with CMF (0.014 vs 0.009% ID $cm^{-3}$, $P < 0.05$). However, uptake of In-111 was less also among patients treated with three cycles of FEC and high-dose chemotherapy than among those given nine cycles of dose-escalated FEC ($P < 0.05$), and there was no difference in the mean cardiac uptake between patients treated with high-dose chemotherapy and those given CMF ($P > 0.1$).

Similar results were obtained, when the HLRs were calculated (Figure 1). The HLRs were large in group I (median 1.98; range 1.36–2.24) as compared to group II (median 1.51; range 1.20–1.82; $P < 0.001$) or to group III (median 1.44; range 1.15–1.68; $P < 0.001$). There was no significant difference in the HLRs between patients treated with high-dose therapy or CMF. The HLR was higher than the chosen cut-off level of 1.58 in 13 (93%) out of the 14 patients in group I, six (43%) out of the 14 patients in group II, and in one (13%) of the eight patients in group III.

The association between the HLRs and the cumulative dose of epirubicin received is shown in Figure 2. The data fits with a linear regression model, the higher the epirubicin dose given, the higher the uptake ($R^2 = 0.59$, $P < 0.001$). No correlation was found between the HLRs and the time elapsed since the last dose of chemotherapy, or between the HLRs and the estimated absorbed cardiac dose of radiation.

### DISCUSSION

In the present study almost all patients treated with dose-escalated FEC had cardiac antimyosin uptake that was considered as pathological, whereas only one patient treated with i.v. CMF had such findings. Moreover, the only patient in the dose-escalated FEC group with a HLR considered as normal had received the lowest cumulative dose of epirubicin in this group of patients (502 mg $m^{-2}$). Interestingly, the cardiac uptake of the patients treated with three cycles of conventional FEC and high-dose CTCb differed only marginally from the uptakes measured in patients treated with CMF, suggesting that both a short course of conventional FEC and a single course of CTCb are less cardiotoxic as compared with the dose-escalated FEC. The limited subclinical cardiotoxicity found among patients treated with high-dose CTCb 8–36 months after therapy suggests that long-term cardiotoxicity related to alkylating agents even if given in high single doses may not be of a major concern, at least in comparison to moderately high peak doses of epirubicin. However, the clinical consequences of subclinical cardiotoxicity detected by antimyosin antibody scintigraphy remain uncertain and may require long-term follow-up of the patients.

The biological action of the anthracyclines includes inhibition of the mitochondrial oxidative phosphorylation, DNA and RNA polymerases and DNA repair enzymes, the metallothionein synthesis, topoisomerase II and the helicases. Further effects occur by the production of free radicals, membrane modulation and endonucleolytic cleavage (Booser and Hortobagyi, 1994). Epirubicin-induced pathological changes in the human myocardium are similar to those of doxorubicin, such as dilation of the sarcoplasmic reticulum, myofibrillar loss and increase in interstitial fibrosis without inflammatory cells (Torti et al, 1986).

Although the extent of cardiotoxic damage can be most accurately determined by endomyocardial biopsy, the invasive nature of the method limits its use in clinical practice. Serial left ventricular ejection fraction measurements, although controversial, are the most widely used method for cardiac monitoring during chemotherapy administration. In-111 antimyosin monoclonal antibody scan is also minimally invasive, and it has been successfully used in several clinical trials to detect acute doxorubicin cardiotoxicity (Estorch et al, 1993; Carrio et al, 1993, 1995; Valdes et al, 1994).

Binding of this antibody to intracellular myosin takes place only after sarcolemmal disruption is present. Only cardiac and skeletal myosin show high specificity to antimyosin antibody (Kairemo et al, 1990; Bhattacharya and Lahiri, 1991), and, therefore, also extracardial lesions may be detected (Kairemo et al, 1996). The myocardial uptake of In-111 antimyosin monoclonal antibody correlates well with the pathologic grade of the cell damage in myocarditis (Yamada et al, 1990). Moreover, pathologic

© 2000 Cancer Research Campaign

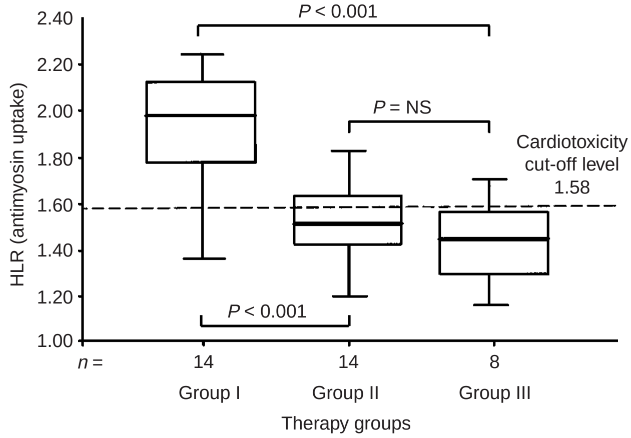

**Figure 1** Distribution of cardiac In-111-antimyosin antibody uptake (heart-to-lung ratio) values by the type of adjuvant therapy given (groups I, II and III, see text; NS, non-significant)

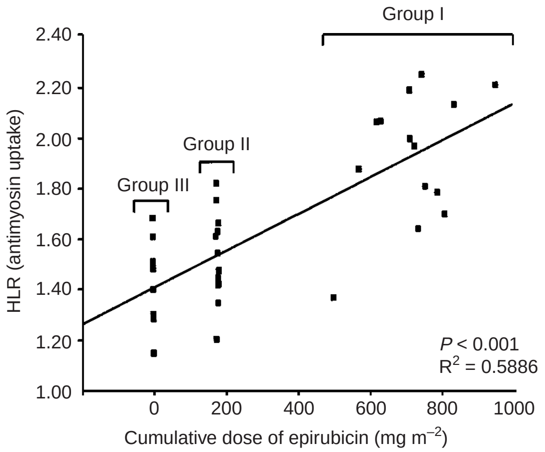

**Figure 2** Association between cardiac In-111-antimyosin antibody uptake (heart-to-lung ratio) and the cumulative dose of epirubicin given (mg $m^{-2}$)

myocardial antimyosin antibody uptake has been observed long after the onset of myocardial cell damage. Yamada et al (1992) demonstrated positive antimyosin uptake in 71% of patients with a myocardial infarction 1.5–12 months after the incident by In-111-antimyosin scans.

In the present study the amount of subclinical cardiac damage paralleled the cumulative epirubicin dose given. As with doxorubicin, congestive heart failure following administration of epirubicin is well documented in the literature (Nielsen et al, 1990; Lopez et al, 1998). Jensen and collaborators (1996) found severe chronic cardiac failure in nearly 10% of patients with breast cancer treated with epirubicin. In their series, cardiac failure developed at a median of 2.5 months after the last dose of epirubicin. As with doxorubicin, the incidence of epirubicin-induced cardiac failure is greatly dependent on the cumulative dose of epirubicin, but it depends also on other factors, such as the mode of administration of epirubicin, concomitant medication, presence of previous cardiac disease, amount of cardiac irradiation and individual and unrecognized factors. Congestive cardiac failure is rare at cumulative epirubicin doses less than 1000 mg $m^{-2}$ in patients without known risk factors for cardiac failure (Jain et al, 1985; Dardir et al, 1989; Nielsen et al, 1990). However, no studies have yet addressed the clinical significance of subclinical cardiac damage, which may be a highly relevant issue in the long-term follow-up.

Cyclophosphamide may also be cardiotoxic, and it may add to the toxicity caused by anthracyclines especially when given in very high doses (Fraiser et al, 1991). Echocardiographic studies have found that left ventricular dysfunction occurs in over 50% of patients undergoing transplantation, and cyclophosphamide cardiotoxicity may be a lethal complication of high-dose therapy (Goldberg et al, 1986; Braverman et al, 1991). However, in the present series the cumulative dose of cyclophosphamide was approximately similar in groups I and II, but the extent of subclinical cardiotoxicity differed considerably between the groups, suggesting that the cumulative dose of cyclophosphamide was not a major determinant for post-transplantation cardiotoxicity. Cardiotoxicity of fluorouracil is low, and the differences in the cumulative doses of fluorouracil are unlikely to explain the present results. The amount of cardiac irradiation was minimal in all three groups. Hence, epirubicin is the likely candidate to explain the increased cardiac uptake of the antimyosin antibody.

We conclude that the majority of patients treated with dose-escalated epirubicin show increased uptake of In-111-labelled antimyosin antibody compatible with subclinical cardiotoxicity. Similar high uptake was not found among patients treated with three cycles of conventional FEC and high-dose chemotherapy containing alkylating agents, suggesting that cardiotoxicity of these treatments is small when assessed a few months to a few years after treatment as compared with nine cycles of dose-escalated FEC, and may not be substantially greater than that of intravenously given CMF. Premature adoption of adjuvant regimens consisting of high cumulative doses or high peak doses of anthracyclines given without cardioprotective drugs may abrogate the survival benefits possibly associated with such regimens, and we recommend that clinical trials with long-term follow-up data are available before adjuvant regimens using high anthracycline doses are widely used.

### REFERENCES

Antman KH, Rowlings PA, Vaughan WP, Pelz CJ, Fay JW, Fields KK, Freytes CO, Gale RP, Hillner BE, Holland HK, Kennedy MJ, Klein JP, Lazarus HM, McCarthy PJ Jr, Saez R, Spitzer G, Stadtmauer EA, Williams SF, Wolff S, Sobocinski KA, Armitage JO and Horowitz MM (1997) High-dose chemotherapy with autologous hematopoietic stem-cell support for breast cancer in North America. *J Clin Oncol* **15**: 1870–1879

Astorri E, Contini GA, Fiorina P, Gavaruzzi D and Fesani F (1996) Myocardial indium-111 antimyosin uptake after uncomplicated coronary artery bypass surgery. *Int J Cardiol* **55**: 239–244

Ballester M, Marti V, Carrio I, Obrador D, Moya C, Pons-Llado G, Berna L, Lamich R, Aymat MR, Barbanoj M, Guardia J, Carreras F, Udina C, Auge JM, Marrugat J, Permanyer G and Caralps-Riera JM (1997*a*) Spectrum of alcohol-induced myocardial damage detected by indium-111-labeled monoclonal antimyosin antibodies. *J Am Coll Cardiol* **29**: 160–167

Ballester M, Marti V, Obrador D, Carrio I and Marrugat J (1997*b*) Role of 111 In-monoclonal antimyosin antibodies in risk stratification of patients with dilated cardiomyopathy referred for heart transplantation. *Transplant P* **29**: 589–591

Bengel FM, Feistel H, Moshage W, Bachman K and Wolf F (1997) Myocardial damage assessed by indium-111-antimyosin: correlation with persistent enteroviral ribonucleic acid in dilated cardiomyopathy. *Eur J Nucl Med* **24**: 1128–1131

© 2000 Cancer Research Campaign

Bergh J, Wiklund T, Erikstein B, Fornander T, Bengtsson NO, Malmstrom P, Kellokumpu-Lehtinen P, Anker G, Bennmarker H, Wilking N (1998) Dosage of adjuvant G-CSF (filgrastim); supported FEC polychemotherapy based on equivalent haematological toxicity in high-risk breast cancer patients. *Ann Oncol* **9**: 403–411

Bhattacharya S, Lahiri A (1991) Clinical role of indium-111 antimyosin imaging. *Eur J Nucl Med* **18**: 889–895

Booser DJ, Hortobagyi GN (1994) Anthracycline antibiotics in cancer therapy. Focus on drug resistance. *Drugs* **47**: 223–228

Braverman AC, Antin JH, Plappert MT, Cook EF, Lee RT (1991) Cyclophosphamide cardiotoxicity in bone marrow transplantation: a prospective evaluation of new regimens. *J Clin Oncol* **9**: 1215–1223

Carrio I, Lopez-Pousa A, Estorch M, Duncker D, Berna L, Torres G, de Andres L (1993) Detection of doxorubicin cardiotoxicity in patients with sarcomas by indium-111-antimyosin monoclonal antibody studies. *J Nucl Med* **34**: 1503–1507

Carrio I, Estorch M, Berna L, Lopez-Pousa J, Tabernero J, Torres G (1995) Indium-111-antimyosin and iodine-123-MIBG studies in early assessment of doxorubicin cardiotoxicity. *J Nucl Med* **36**: 2044–2049

Dardir MD, Ferrans VJ, Mikhael YS, el-Grindy MS, el-Aasar AB, el-Zawahry, Alling DW, Banks SM, el-Mawla NG (1989) Cardiac morphologic and functional changes induced by epirubicin chemotherapy. *J Clin Oncol* **7**: 947–958

Early Breast Cancer Trialists' Collaborative Group (1998) Polychemotherapy for early breast cancer: an overview of the randomised trials. *Lancet* **352**: 930–942

Estorch M, Carrio I, Martinez-Duncker D, Berna L, Torres G, Alanso C, Ojeda B (1993) Myocyte cell damage after administration of doxorubicin or mitoxantrone in breast cancer patients assessed by indium 111 antimyosin monoclonal antibody studies. *J Clin Oncol* **11**: 1264–1268

Fraiser LH, Kanekal S, Kehrer JP (1991) Cyclophosphamide toxicity. Characterising and avoiding the problem. *Drugs* **42**: 781–795

Goldberg MA, Antin JH, Guinan EC, Rappeport JM (1986) Cyclophosphamide cardiotoxicity: an analysis of dosing as a risk factor. *Blood* **68**: 1114–1118

Grathwohl A, Hermans J, Baldomero H (1997) Blood and marrow transplantation activity in Europe 1995. *Bone Marrow Transpl* **19**: 407–419

Green M (1998) Anthracycline cardiotoxicity, no longer an issue? *Ann Oncol* **9**: 691–693

Jain KK, Casper ES, Geller NL, Hakes TB, Kaufman RJ, Currie V, Schwartz W, Cassidy C, Petroni GR, Young CW, Wittes RE (1985) A prospective randomized comparison of epirubicin and doxorubicin in patients with advanced breast cancer. *J Clin Oncol* **3**: 818–826

Jensen BV, Nielsen SL, Skovsgaard T (1996) Treatment with angiotensin-converting-enzyme inhibitor for epirubicin-induced dilated cardiomyopathy. *Lancet* **347**: 297–299

Kairemo KJA, Wiklund TA, Liewendahl K, Miettinen M, Heikkonen JJ, Virkkunen P, Aronen HJ, Blomqvist CP (1980) Imaging of soft-tissue sarcomas with Indium-111-labelled monoclonal antimyosin fab fragments. *J Nucl Med* **31**: 23–31

Kairemo KJA, Blomqvist CP, Miettinen M (1996) Cardiac myxomas. *N Engl J Med* **334**: 1407–1408

Legha S, Benjamin RS, Mackay B (1982) Reduction of doxorubicin toxicity by prolonged intravenous infusion. *Ann Intern Med* **96**: 133–139

Lopez M, Vici P, Di Lauro L, Conti F, Paoletti G, Ferraironi A, Sciuto R, Giannarelli D, Maini CL (1998) Randomized prospective clinical trial of high-dose epirubicin and dexrazoxane in patients with advanced breast cancer and soft tissue sarcomas. *J Clin Oncol* **16**: 86–92

Nielsen D, Jensen JB, Dombernowsky P, Munck O, Fogh J, Brynjolf I, Havsteen H, Hansen M (1990) Epirubicin cardiotoxicity: a study of 135 patients with advanced breast cancer. *J Clin Oncol* **8**: 1806–1810

Peters WP (1996) High-dose chemotherapy for breast cancer. In: *Diseases of the Breast*, pp. 735–743. Lippincott-Raven: Philadelphia

Schutz A, Breuer M, Kemkes BM (1997) Antimyosin antibodies in cardiac rejection. *Ann Thorac Surg* **63**: 578–581

Torti FM, Bristow MM, Lum BL, Carter SK, Howes AE, Aston DA, Brown BW Jr, Hannigan JF Jr, Meyers FJ, Mitchell EP (1986) Cardiotoxicity of epirubicin and doxorubicin: assessment by endomyocardial biopsy. *Cancer Res* **46**: 3722–3727

Valdes ORA, Bokkel Huinink WW, Hoeve RF, van Tinteren H, Bruning PF, van Vlies B, Hoefnagel CA (1994) Usefulness of indium-111 antimyosin scintigraphy in confirming myocardial injury in patients with anthracycline-associated left ventricular dysfunction. *Ann Oncol* **5**: 617–622

Verna E, Ceriani L, Casucci R, Repetto S, Roncari G, Binaghi G (1995) Myocardial uptake of indium-111 antimyosin after coronary angioplasty. Relationship with the total burden of ischaemia. *Eur Heart J* **16**: 478–484

Yamada T, Matsumori A, Watanabe Y, Tamaki N, Yonekura Y, Endo K, Konishi J, Kawai C (1990) Pharmacokinetics of indium-111-labeled antimyosin monoclonal antibody in murine experimental viral myocarditis. *J Am Coll Cardiol* **16**: 1280–1286

Yamada T, Tamaki N, Morishima S, Konishi J, Yoshida A, Matsumori A (1992) Time course of myocardial infarction evaluated by indium-111-antimyosin monoclonal antibody scintigraphy: clinical implications and prognostic value. *J Nucl Med* **33**: 1501–1508

© 2000 Cancer Research Campaign
